# A randomized controlled pilot study of the effectiveness of magnolia tea on alleviating depression in postnatal women

**DOI:** 10.1002/fsn3.1442

**Published:** 2020-02-10

**Authors:** Lili Xue, Jie Zhang, Huaxiang Shen, Ling Ai, Rongrong Wu

**Affiliations:** ^1^ Department of Obstetrics Jiaxing University Affiliated Women and Children Hospital Jiaxing China; ^2^ Department of Hepatobiliary Surgical First Hospital of Jiaxing Jiaxing China; ^3^ Department of Laboratory Jiaxing University Affiliated Women and Children Hospital Jiaxing China

**Keywords:** complementary therapy, herbal tea, magnolia tea, postpartum depression, quality of sleep

## Abstract

The magnolia tea has been used in traditional oriental medicine for multiple purposes including sleep aid. Postpartum depression is a mental illness that adversely affects the health and well‐being of many families with newborns. Given the known effectiveness and relative safety, herein we aimed to investigate whether magnolia tea has a palliative effect on postpartum depression. The qualified participants were randomly assigned to the intervention group or the control group. The participants in the intervention group drunk magnolia tea, while the control group received regular postpartum care only. The outcome variables including Postpartum Sleep Quality Scale (PSQS), Edinburgh Postnatal Depression Scale (EPDS), and Postpartum Fatigue Scale (PFS) were assessed and compared. In comparison with the control group, the intervention group demonstrated significant difference for physical‐symptom‐related sleep inefficiency (PSQS Factor 2) at 3 weeks post‐test (*t* = −2.10, *p* = .03). The comparison results also revealed significant differences for PFS at both 3 weeks post‐test (*t* = −2.02, *p* = .04) and 6 weeks post‐test (*t* = −1.99, *p* = .04). Further, magnolia tea intervention significantly alleviated the symptoms of depression, reflected by the EPDS scores at 3 weeks post‐test (*t* = −2.38, *p* = .02) and 6 weeks post‐test (*t* = −2.13, *p* = .02). Our trial results suggested that drinking single‐ingredient magnolia tea for a 3‐week duration has positive effects on postpartum women. Magnolia tea is recommended as a supplementary approach to ameliorate sleep quality of postpartum women, while alleviating their symptoms of depression.

## Graphical abstract

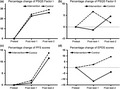

## 1 INTRODUCTION

Postpartum depression (PPD), also known as postnatal depression, is a mental illness associated with childbirth. PPD adversely affects the health and well‐being of many new mothers, the newborn infants, as well as their families (Yim, Tanner Stapleton, Guardino, Hahn‐Holbrook, & Dunkel Schetter, 2015). Psychological symptoms of PPD include grief, depression, anxiety, intermittent crying, and irritating sensitivity (Ko, Chang, & Chen, 2010). In addition, postpartum women experience worse quality of sleep due to the abnormal wake–sleep cycle and prolonged night wake time during the first 5 postpartum weeks (Shinkoda, Matsumoto, & Park, 1999). Sleep quality is important for the psychophysiological health of postpartum women. It has been reported that women with postpartum depression experience more frequent sleep disturbances, less total sleep time, and lower sleep efficiency than women who have no depression during pregnancy or postpartum, especially at the first 3 months after delivery (Lee, Zaffke, & McEnany, 2000). Sleep deprivation during the postpartum period may develop into chronic insomnia, which could produce consistent mood symptoms as PPD (Dorheim, Bondevik, Eberhard‐Gran, & Bjorvatn, 2009). Other significant consequences may include maternal–infant bonding issues, or the behavioral or emotional difficulties in the infant (Okun, 2015).

It is essential to prevent maternal postpartum depression by taking appropriate intervention. A great number of herbal teas have been used for sleep aids, such as St. John's wort, passion flower, chamomile, valerian, and Kava (Beaubrun & Gray, 2000). Recently, reports have shown that herbal teas can be used for PPD recovery treatment, but the duration of action is limited. For instance, chamomile tea intervention lowered immediate physical‐symptom‐related sleep inefficiency and depression in postnatal women, but has no effects during the 4 weeks post‐test (Chang & Chen, 2016).

Magnolia is a tree (*magnolia officinalis*) native to China. The magnolia plant has an ancient history as a therapeutic compound in traditional oriental medicine. Both the bark of the magnolia tree and its flowers are used therapeutically. Modern pharmacological approaches revealed that magnolia bark extracts have antioxidant, anti‐inflammatory, and anti‐tumor properties. Among other conditions, magnolia extracts possess anti‐anxiety and neuroprotective activities (Ge et al., 2017). The increasing interests in the use of herbal remedies by patients require mental health professionals to explore more effects of those commonly used herbs. Recently, magnolia bark extracts have been recognized as a food additive worldwide.

Given its sedative effects and relative safety, the purpose of this study was to investigate whether magnolia tea has a palliative effect on postpartum depression. Specifically, a randomized controlled trial was performed to compare the immediate and long‐term effects at 3 and 6 weeks postpartum of the magnolia tea therapy on variable postnatal outcomes on the scales of sleep quality, fatigue, and depression.

## 2 MATERIALS AND METHODS

### 2.1 Study participants

The study was approved by the Ethical Committee of Jiaxing University Affiliated Women and Children Hospital. Written consents were obtained from all participants prior to the study. The cohort was enrolled between 2015 and 2018. A total of 143 postpartum women were recruited. The parameters of the clinical health conditions and demographic data of the participants were retrieved from their electronic medical records archived in the hospital database. The inclusion criteria were as follows: (a) Postpartum Sleep Quality Scale (PSQS, described in details later) score ≥ 16, (b) with normal childbirth, and (c) no postnatal complications. Postnatal women who had no interest in the study, disliked the herbal tea, or moved elsewhere were further excluded.

### 2.2 Study design

Following the initial recruitment, patients were randomized into intervention group and control group with matched demographic characteristics. For the intervention group, in addition to the normal postpartum care provided by the hospital, patients were directed to drink one cup of magnolia tea (origin: China) daily for 3 consecutive weeks. The tea was prepared by soaking the tea in 300 ml of hot water for 12 min. In contrast, patients in the control group did not receive any herbal tea intervention.

### 2.3 Outcome measurements

The modified Postpartum Sleep Quality Scale (PSQS) containing 14 items (Yang, Yu, & Chen, 2013) was used to assess the sleep quality of the subjects. This PSQS is composed of two main categories: physical‐symptom‐related sleep inefficiency and infant night‐care‐related daytime dysfunction. Briefly, patients were required to rate each sleep problem item during the first 2 weeks on a 5‐point Likert scale (never = 0, few = 1, sometimes = 2, often = 3, almost always = 4). The total possible scores ranged from 0 to 56, while scores are negatively related to sleep quality.

Furthermore, we used the Edinburgh Postnatal Depression Scale (EPDS) containing 10 short statements (Cox, Holden, & Sagovsky, 1987) to evaluate postpartum depression severity of the participants. The patients were screened by answering how they felt in the past 7 days. After completing all 10 items, and the numbers were added up to find the total score. Responses are scored 0, 1, 2, and 3 based on the seriousness of the symptom, and mothers scoring above 12 or 13 are likely to be suffering from depression. To minimize the limited English reading difficulties, a validated Chinese version of the EPDS with appropriate linguistic and cultural contexts was utilized in the study (Heh, 2001), and the patients were able to complete the scale personally.

In addition, the 12‐item Postpartum Fatigue Scale (PFS) was used to assess postpartum fatigue via a structured, self‐report questionnaire. Patients were required to evaluate and score their level of fatigue self‐perceivably during the past week on a 4‐point Likert scale, while none = 0, mild = 1, moderate = 2, and severe = 3. The total score ranges from 0–36 by adding up all scores in each question, while a higher score reflects higher postpartum fatigue level.

### 2.4 Statistical analysis

All statistical analyses in this trial were conducted using SPSS statistical software package (version 20.0). Demographic characteristics were summarized using descriptive statistics. Data were expressed as percentage, mean and standard deviation (mean ± *SD*). The independent *t* test and Fisher's exact test were perfume to compare the significance. Two‐sample *t* test was used to examine the mean differences in the outcome variables including PSQS, EPDS, and PFS factors. The percentage changes of outcomes from pretest to post‐test in the intervention and control groups were also demonstrated. *p* < .05 was considered statistically significant.

## 3 RESULTS

### 3.1 Investigation flow

**Figure 1:**
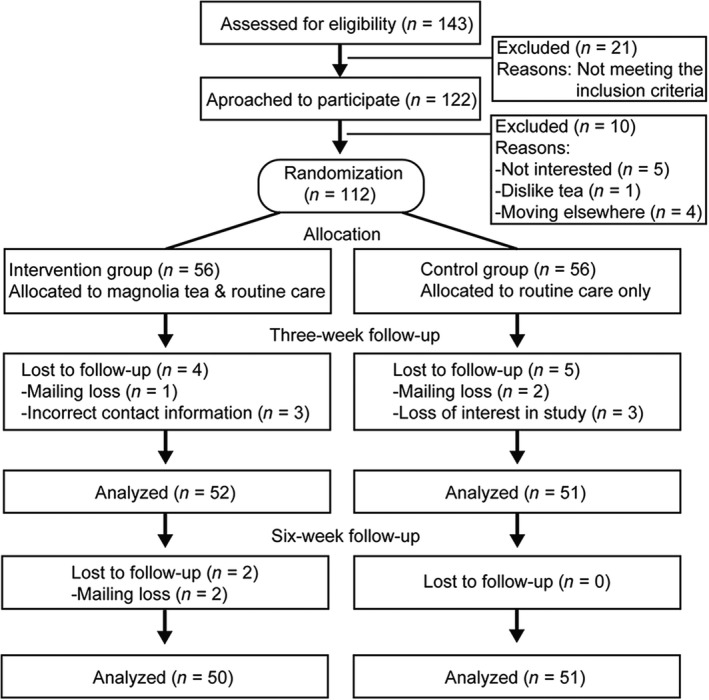
Flow diagram of this investigation

As shown in the flow diagram (Figure 1), a total of 143 postnatal women were enrolled in this study. There were 21 patients excluded from being approached since they did not meet the inclusion criteria. Further, another 10 patients were excluded due to variable reasons, while 5 of them showed no interest in the study, 1 person does not drink herbal tea, and 4 of them moved to other places. The remaining 112 participants were randomly assigned into intervention group and control group (*n* = 56, respectively). In the intervention group, 4 participants were lost to follow‐up due to mailing loss or incorrect contact information. In the control group, 5 participants were lost to follow‐up due to mailing loss or study interest loss. Eventually, valid data were analyzed from 52 participants in the intervention group and 51 participants in the control group for 3 weeks post‐test. At the 6 weeks post‐test, we lost mailing of 2 patients in the intervention group, while all remaining patients in the control group stayed in the study.

### 3.2 Demographic characteristics

As demonstrated in Table 1, no significant difference was observed between the invention group and control group in terms of demographic characteristics including age (25.6 vs. 25.3 years, *p* = .9), marital status (94.2% vs. 96.1%, *p* = .9), education level, occupation, household income, type of delivery, parity, gender of newborn, gestational age of new born, prenatal education, support from families for parenting, and type of infant feeding.

**Table 1:** Comparison of demographic characteristics between invention and control groups

Variables	Total (*n* = 103)	Invention group (*n* = 52)	Control group (*n* = 51)	*χ* ^2^	*p*
Age					
Mean (*SD*)	25.4 (4.6)	25.6 (4.3)	25.3 (4.4)	0.05^a^	.96
Range	20–37	20–37	20–36		
Marital status					
Married	98 (95.1%)	49 (94.2%)	49 (96.1%)	0.19	.99
Single/others	5 (4.9%)	3 (5.8%)	2 (3.9%)		
Education level					
Primary/secondary school	29 (28.2%)	14 (27.0%)	15 (29.4%)	0.04^b^	.98
College/university	58 (56.3%)	30 (57.7%)	28 (54.9%)		
Graduate school	16 (15.5%)	8 (15.3%)	8 (15.7%)		
Occupation					
Employed	93 (90.3%)	46 (88.5%)	47 (92.2%)	0.24	.71
Not‐working	10 (9.7%)	6 (11.5%)	4 (7.8%)		
Household income (monthly)					
Lower than $750	31 (30.1%)	17 (32.7%)	14 (27.4%)	0.85	.58
$750 ~ $1,500	57 (55.3%)	27 (51.9%)	30 (58.9%)		
$1,500 & above	15 (14.6%)	8 (15.4%)	7 (13.7%)		
Type of delivery					
Vaginal	58 (56.3%)	27 (52.0%)	31 (60.8%)	1.01	.46
Cesarean	45 (43.7%)	25 (48.0%)	20 (39.2%)		
Parity					
Primiparous	82 (79.7%)	44 (84.6%)	38 (74.5%)	1.47	.39
Multiparous	21 (20.3%)	8 (15.4%)	13 (25.5%)		
Gender of newborn					
Male	54 (52.4%)	25 (48.1%)	29 (56.9%)	0.54	.69
Female	49 (47.6%)	27 (51.9%)	22 (43.1%)		
Gestational age of newborn					
Term	86 (83.5%)	45 (86.5%)	41 (80.4%)	0.11	.91
Preterm	17 (16.5%)	7 (13.5%)	10 (19.6%)		
Prenatal education					
Accepted	20 (19.4%)	11 (21.2%)	9 (17.7%)	0.12	.88
Did not accept	83 (80.6%)	41 (78.8%)	42 (82.3%)		
Support from families for parenting					
Accepting	62 (60.2%)	28 (53.8%)	34 (66.7%)	4.212	.127
Not accepting	41 (39.8%)	24 (46.2%)	17 (33.3%)		
Type of infant feeding					
Breast	59 (57.3%)	28 (53.8%)	31 (60.8%)	3.543	.156
Bottle	5 (4.8%)	1 (2.0%)	4 (7.8%)		
Mixed	39 (37.9%)	23 (44.2%)	16 (31.4%)		

a Independent *t* test.

b Fisher's Exact test.

### 3.3 Assessment of postnatal depression outcomes

As illustrated in Table 2, all the outcome variables did not show significant differences prior to the magnolia tea intervention. We run a two‐sample *t* test to compare the effects of the magnolia tea intervention. The infant night‐care‐related daytime dysfunction (PSQS Factor 1) did not show significant difference at 3 weeks post‐test (*t* = −0.05, *p* = .96) and 6 weeks post‐test (*t* = −0.39, *p* = .72). Notably, we observed significant difference for physical‐symptom‐related sleep inefficiency (PSQS Factor 2) at 3 weeks post‐test (*t* = −2.10, *p* = .03), but not at 6 weeks post‐test (*t* = −0.35, *p* = .75). The comparison results also revealed significant differences for PFS at both 3 weeks post‐test (*t* = −2.02, *p* = .04) and 6 weeks post‐test (*t* = −1.99, *p* = .04). For the EPDS outcome, magnolia tea intervention significantly alleviated the symptoms of depression, reflected by the EPDS scores at 3 weeks post‐test (*t* = −2.38, *p* = .02) and 6 weeks post‐test (*t* = −2.13, *p* = .02).

**Table 2:** Two‐sample *t* test for outcome variables

Outcome variables	Invention group Mean (*SD*)	Control group Mean (*SD*)	*t*	*p*
PSQS Factor 1				
Pretest^c^	19.79 (4.132)	19.58 (3.762)	0.067	.951
3 weeks post‐test	14.97 (3.970)	15.16 (4.530)	−0.056	.962
6 weeks post‐test	14.05 (4.032)	14.48 (3.912)	−0.398	.729
PSQS Factor 2				
Pretest	8.03 (2.980)	8.51 (3.421)	−0.432	.653
3 weeks post‐test	7.50 (3.231)	8.62 (2.783)	−2.103	.030
6 weeks post‐test	7.88 (3.124)	8.12 (3.534)	−0.357	.754
PFS				
Pretest	15.09 (7.312)	15.97 (7.446)	−0.639	.442
3 weeks post‐test	14.79 (6.809)	15.83 (7.551)	−2.021	.045
6 weeks post‐test	13.01 (5.890)	14.13 (6.039)	−1.991	.048
EPDS				
Pretest	9.67 (4.089)	9.43 (4.853)	0.981	.253
3 weeks post‐test	9.42 (4.341)	10.54 (4.765)	−2.389	.020
6 weeks post‐test	8.77 (3.942)	9.79 (4.467)	−2.134	.027

Note

PSQS Factor 1: infant night‐care‐related daytime dysfunction. PSQS Factor 2: physical‐symptom‐related sleep inefficiency.

### 3.4 Comparison of percentage change of outcomes over time

**Figure 2:**
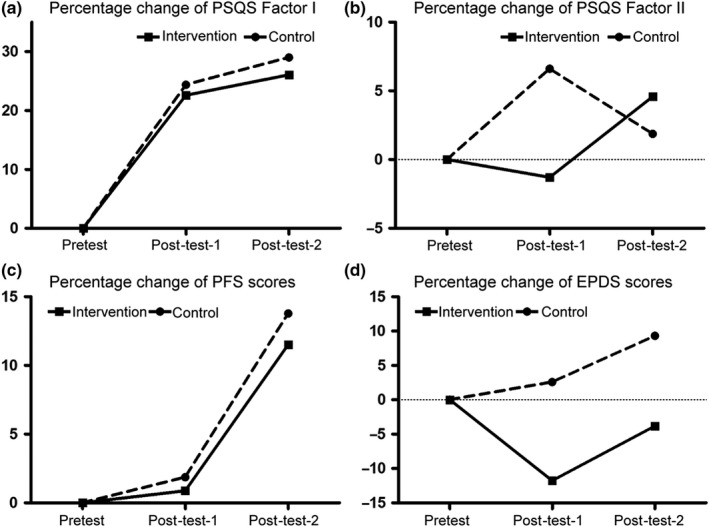
Percentage changes of outcomes from pretest to post‐tests

In order to trace the dynamic effects of the magnolia tea intervention over time, the graphical representation of the percentage change in the four outcome variables was presented in Figure 2. In panel A, we can see the positive increases of PSQS Factor I in both intervention and control groups, identifying no improvement of postpartum sleep quality. In panel B, the magnolia tea intervention group showed obvious negative percentage change at 3 weeks post‐test, indicating the improvement of physical‐symptom‐related sleep inefficiency. Further, we observed negative percentage changes of PFS scores in both groups at both 3 and 6 weeks post‐tests, suggesting less severe postpartum fatigue over time. Interestingly, the changes in the magnolia tea intervention group were larger than the ones in the control group (Figure 2c), suggesting the positive intervention of magnolia tea in lowering the fatigue. Similarly, EPDS score in the intervention group was largely lowered at 3 weeks post‐test, indicating the improvement of postnatal depression symptoms, and this protective effect remained at the 6 weeks post‐test (Figure 2d). Intriguingly, the postnatal depression extent was even higher at post‐tests when compared with pretest, further providing support for essentiality of postnatal depression management.

## 4 DISCUSSION

Postpartum depression is a complication of giving birth, which influences more than 10% of women who experience an episode of major or minor depression within the first three postpartum months (Marcus, Flynn, Blow, & Barry, 2003). These women might try hard to cope with their babies and other household errands, but their enjoyments of life are seriously sacrificed. Recent studies have consistently showed the deleterious effects of postpartum depression on the emotional and cognitive development of the infants, and later childhood (Zhu et al., 2014). Given the public health relevance, the prevention of PPD holds tremendous expectations for alleviating the illness burdens and life quality of postnatal women. In this study, we evaluated the effects of intervention effects of singe‐ingredient magnolia tea on prenatal women using three independent instruments including PSQS, EPDS, and PFS. Our results showed that drinking magnolia tea for 3 weeks significantly improved the sleep quality, and alleviated extents of fatigue and depression in postpartum women at both 3 and 6 weeks post‐tests, suggesting that magnolia tea could serve as a relatively safe and cost‐effective supplementation to postpartum women.

There were 143 patients enrolled at the beginning of our study. According to the independent *t* test based power analysis, the sample size was large enough to achieve a power of 0.80 with a 0.05 alpha (Sawilowsky & Hillman, 1992). There were 112 valid patients remained in the study for data analysis, with 56 postnatal women in the intervention group and control group. The dropout rate was similar as expected 20%. Despite the achievement of the statistical power, the duration of the cohort study was almost 3 years, and we acknowledge that more efforts could have been paid to enlarge the sample size to get more thorough analysis on the effectiveness and side effects of magnolia tea. In addition, the present study only included 56 Chinese postnatal women, the physiological characteristics of other patients with different genetic background might bring new concerns prior to considering this supplementary herbal tea.

Three different validated instruments were used to assess the outcomes in this study. To minimize the limited English reading difficulties, a validated Chinese version of these instruments were used in the study. In addition, during the data collection, all data were double‐checked carefully to eliminate data entry inaccuracies. The scales of these measurement can be completed in 10 min with a simple scoring method. The baseline data of both the control and intervention groups were obtained at pretests, and the demographic characteristics in both groups were well matched to minimize baseline variables. To be noted, the participants in the control group were not given magnolia tea. Nevertheless, we also did not require the diet information of the participants for further analysis because the other diet components among the participants could interfere the observed effects of magnolia tea. To minimize this interference, the participants were assigned to the control and tea groups randomly, and we still observed the significant difference with magnolia tea intervention.

Our data showed that magnolia tea work effectively to promote sleep and alleviate depression. We observed significant amelioration for physical‐symptom‐related sleep inefficiency (PSQS Factor 2) at 3 weeks post‐test (*t* = −2.10, *p* = .03). This could be attributed to the improvement of PFS, which was alleviated by magnolia tea intervention, as shown at 3 weeks post‐test (*t* = −2.02, *p* = .04) and 6 weeks post‐test (*t* = −1.99, *p* = .04). Interestingly, the magnolia tea intervention did not impact PSQS Factor 1, infant night‐care‐related daytime dysfunction. We speculated that the enjoyment of life change by newborn balanced the psychological attacks. To be noted, the effects of magnolia tea on PSQS Factor 2 disappeared at 6 weeks post‐test, suggesting the limitation of magnolia tea. However, magnolia tea intervention significantly improved the symptoms of depression, reflected by the EPDS scores at 3 weeks post‐test (*t* = −2.38, *p* = .02) and 6 weeks post‐test (*t* = −2.13, *p* = .02). Compared with the study on the chamomile tea intervention in prenatal depression (Chang & Chen, 2016), magnolia tea demonstrated longer duration on the post‐tests, suggesting that magnolia tea could be another comparable or even superior supplemental than chamomile tea.

The anxiolytic activity of extracts of Magnolia officinalis bark have been studied in different studies (Kalman et al., 2008; Liu et al., 2015). In terms of molecular mechanisms, research shows at least one bioactive compound in magnolia bark can shorten sleep latency and increase the amount of time. An active ingredient, magnolol, has been reported to regulate gamma‐aminobutyric acid receptor A (GABA_A_) receptor, which mediates sleep in humans and animals (Chen et al., 2012; Gottesmann, 2002). Further, as the main inhibitory ligand‐gated ion channels in the adult mammalian central nervous system (CNS), the expression of GABA_A_ receptors reduces anxiety and seizure susceptibility (Maguire, Stell, Rafizadeh, & Mody, 2005). It has been reported that the extract and the main bioactive constituents, including magnolol and honokiol, can activate cannabinoid (CB) receptors (Rempel et al., 2013). CB1 receptor regulates important brain functions by modulating excitatory and inhibitory neurotransmission (Wilson & Nicoll, 2002), and CB2 receptors in dopaminergic neurons play important roles in the modulation of anxiety and depression (Liu et al., 2017). The scientific rationale for the clinical use of honokiol in the treatment of Alzheimer's disease through attenuated amyloid β‐induced cell death has also been reported (Hoi, Ho, Baum, & Chow, 2010). Research indicates that the anxiolytic activity of magnolia is related to neurotransmitters, serotonin, and dopamine (Poivre & Duez, 2017). Briefly, honokiol and magnolol were able to normalize the biochemical abnormalities in brain serotonin via up‐regulating the cyclic adenosine monophosphate pathway (Lee et al., 2011). In vitro binding and functional assays revealed that *Magnolia* bark extracts interact with the adenosine A1 receptor, dopamine transporter, and dopamine D5 receptor, and serotonin receptors (Koetter, Barrett, Lacher, Abdelrahman, & Dolnick, 2009). Taken together, the anxiolytic activity in magnolia tea could be related to the multiple roles in the mediation with the GABA_A_ receptor, cannabinoid receptors, and neurotransmitters.

In spite of the growing evidence of the efficacy of herbal preparations in treating psychiatric conditions, the bench to bedside translation is hampered by the complex chemical compounds of the herbs. Therefore, the lack of standardization in the preparations of single‐ingredient magnolia tea is one of the limitations of this study. The origin and the industrial production process may vary among batches, making difficulty to normalize the results. Since the specific ingredients in the tea extracts which entitled this anti‐depression effects in postnatal women has not been identified, the method could not serve as medical recommendations.

## 5 CONCLUSION

In summary, the magnolia tea intervention on postnatal women was effective in alleviating postpartum depression outcomes and thus could be introduced as supplementary treatment for the postnatal women. Future studies could focus on the standardization of this strategy on broader populations.

Our trial results suggest that drinking single‐ingredient magnolia tea for a 3‐week duration has positive effects on postpartum women. Magnolia tea is recommended as a supplementary approach to ameliorate sleep quality of postpartum women, while alleviating their symptoms of depression.

## CONFLICT OF INTEREST

The authors declare that they do not have any conflict of interest.

## ETHICAL APPROVAL

This study conforms to the Declaration of Helsinki. Written informed consent was obtained from all participants. The study was approved by the Ethical Committee of Jiaxing University Affiliated Women and Children Hospital.
